# REEF: searching REgionally Enriched Features in genomes

**DOI:** 10.1186/1471-2105-7-453

**Published:** 2006-10-16

**Authors:** Alessandro Coppe, Gian Antonio Danieli, Stefania Bortoluzzi

**Affiliations:** 1 Department of Biology, University of Padova, via G. Colombo 3, 35131, Padova, Italy

## Abstract

**Background:**

In Eukaryotic genomes, different features including genes are not uniformly distributed. The integration of annotation information and genomic position of functional DNA elements in the Eukaryotic genomes opened the way to test novel hypotheses of higher order genome organization and regulation of expression.

**Results:**

REEF is a new tool, aimed at identifying genomic regions enriched in specific features, such as a class or group of genes homogeneous for expression and/or functional characteristics. The method for the calculation of local feature enrichment uses test statistic based on the Hypergeometric Distribution applied genome-wide by using a sliding window approach and adopting the False Discovery Rate for controlling multiplicity. REEF software, source code and documentation are freely available at .

**Conclusion:**

REEF can aid to shed light on the role of organization of specific genomic regions in the determination of their functional role.

## Background

In Eukaryotic genomes, different features including genes are not uniformly distributed. The integration of annotation information and genomic position of functional DNA elements in the Eukaryotic genomes opened the way to test novel hypotheses of higher order genome organization and regulation of expression. Different studies attempted at searching genomic regions enriched in specific gene categories and at correlating the localization of genes with their expression characteristics. In this way, clusters of highly expressed genes in the human genome were identified [1,2], whereas frequent co-localization of co-expressed genes was found in the *Drosophila *genome [3] and, in a broad study, in six Eukaryotic genomes [4]. Other studies described chromosomal clustering of muscle expressed genes and of reproductive genes in *Caenorhabditis elegans *[5,6] and chromosomal domains of genes highly expressed in liver and others in colon, containing well conserved genes among human, mice and rat [7]. The reconstruction of the human heart transcriptome showed then that most heart genes are clustered in small groups of elements, which show in their promoters similar regulatory elements and the products of which share more functional features than expected by chance [8]. In these studies, diverse experimental schemas were adopted to search for local clusters of special gene categories and different systematic analyses were carried out by custom-tailored scripts applied on large-scale datasets. As far as program availability, to our knowledge dedicated software for this kind of analysis is still unavailable if we exclude some R functions specifically devoted to the analysis of differential gene expression along the human genome and to detect genomic regions involved in tumor-related imbalances [9-11] or yeast specific software [12]. In this paper we present a novel program, called REEF (Regionally Enriched FEatures), allowing the systematic analysis of any genome for regions that show significant local enrichment of specific features and to display results in the context of genome annotation. REEF scans the considered genome using a sliding window approach and adopts the False Discovery Rate to give a genome-wide significance of observed local enrichment. A feature class may be established as a group of sequences or of genomic positions with specific characteristics. For instance a feature class may be the group of occurrences of a sequence motif in a genome or it may be a set of genes showing a specific expression pattern or a group of genes encoding products involved in a given biological process, in a molecular function, as encoded by Gene Ontology terms. Thus REEF can be used to inspect for local clustering of different kind of features, aiding to shed light on the role of organization of specific genomic regions in the determination of their functional role.

## Implementation

### REEF method

REEF aims at identifying regions of a genome enriched in specific features, as compared with a reference landscape of features density, by using a sliding window approach. REEF takes as input a list of reference features (*RF*; e.g. human genes) mapped to a genomic DNA sequence, a list of features selected (*SF*) among the *RF *(e.g. human genes specifically expressed in a given tissue), along with their genomic positions and the number and the length of chromosomes in the considered genome. Once selected the values for the size of the sliding window and for the shift between adjacent windows, the significance of regional enrichment in *SF *observed in each window, is calculated by using the Hypergeometric Distribution [12]. Given the known distribution of *RF*, a *p-value *is associated to each window, corresponding to the probability of observing by chance a number of *SF *equal or greater than the observed. Let *S *be the total number of *SF *over the entire genome, *R *the total number of *RF *over the entire genome, and *r *the number of *RF *in a given window (with *R *≥ *r *and *S *≥ *r*). The probability of observing by chance at least *k SF *out of *r RF *in the window is the pointwise significance of the observed numbers of *SF *in the window (*p-value*, *p*):

px≥k=∑x=kr(Sx)(R−Sr−x)(Rr)
 MathType@MTEF@5@5@+=feaafiart1ev1aaatCvAUfKttLearuWrP9MDH5MBPbIqV92AaeXatLxBI9gBaebbnrfifHhDYfgasaacH8akY=wiFfYdH8Gipec8Eeeu0xXdbba9frFj0=OqFfea0dXdd9vqai=hGuQ8kuc9pgc9s8qqaq=dirpe0xb9q8qiLsFr0=vr0=vr0dc8meaabaqaciaacaGaaeqabaqabeGadaaakeaacqWGWbaCdaWgaaWcbaGaemiEaGNaeyyzImRaem4AaSgabeaakiabg2da9maaqahabaWaaSaaaeaadaqadaqaauaabeqaceaaaeaacqWGtbWuaeaacqWG4baEaaaacaGLOaGaayzkaaWaaeWaaeaafaqabeGabaaabaGaemOuaiLaeyOeI0Iaem4uamfabaGaemOCaiNaeyOeI0IaemiEaGhaaaGaayjkaiaawMcaaaqaamaabmaabaqbaeqabiqaaaqaaiabdkfasbqaaiabdkhaYbaaaiaawIcacaGLPaaaaaaaleaacqWG4baEcqGH9aqpcqWGRbWAaeaacqWGYbGCa0GaeyyeIuoaaaa@4CAC@

The False Discovery Rate (FDR) [13] is used to circumvent the problem of multiple testing when calculating the genome-wide statistical significance for the observed enrichment in *SF *in a given region. After sorting windows by *p-values *over the entire genome, *q-values *(FDR) are calculated. *Q *(*q-value*) for each window is defined as *Q *= (p*N)/*i*, where *p *is the *p-value *of the window, *N *the total number of windows considered and *i *the number of windows with a *p-value *not higher than *p*. Given a global threshold for the genome wide FDR (e.g. 5%), the number of windows "significantly enriched in *SF*" is determined. The position and length of one cluster of *SF *is defined as the genome coverage of the maximum number of adjacent windows statistically significant.

REEF is an application written in Python [14], which runs both under Microsoft Windows and GNU/Linux. The wxPython multiplatform GUI toolkit is used to provide a graphical user interface. The software and its manual are freely available for download [15] [see additional files 1, 2, 3].

### Display of results

**Figure 1:**
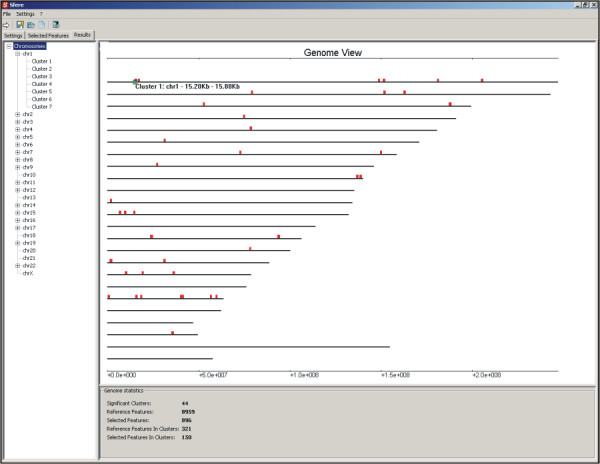
Screenshot of REEF genome view. In the main panel, the positions along human chromosomes of 44 clusters of at least 3 tissue-specific genes are shown; the navigating bar at the left allows to display selected chromosomes or clusters, whereas the bottom panel contains a genomic summary about data and results.

**Figure 2:**
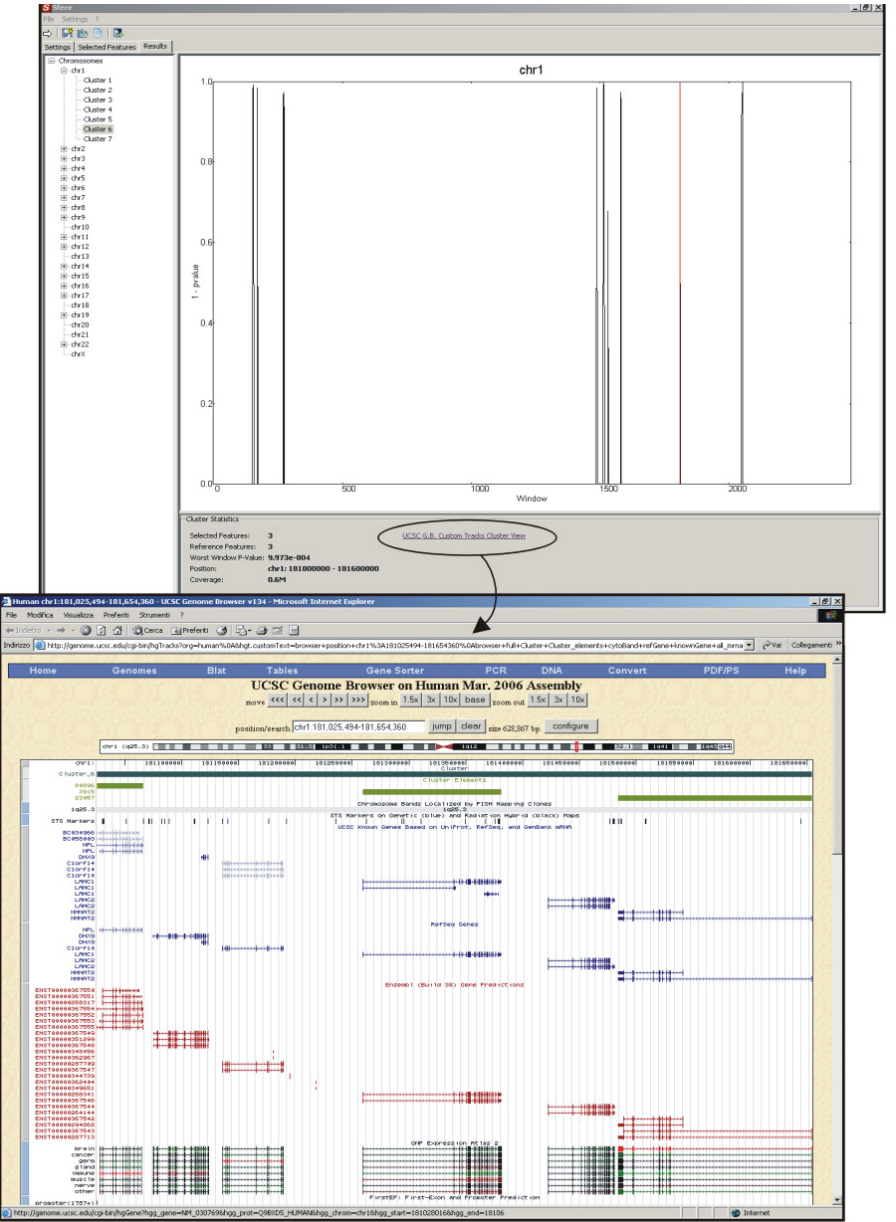
REEF chromosome plot and cluster view. In the chromosome plot at the top of the figure, the enrichment in tissue-specific genes along chromosome 1 is represented by plotting the quantity 1 - *p-value *for subsequent windows considered on the DNA sequence. The value corresponding to the sixth cluster is shown in red and a summary of analyzed data and results for the selected cluster is given below the plot, along with a link to the view in the UCSC Genome Browser of the cluster region with custom annotation tracks. The cluster view is shown in the bottom part of the figure.

Results of statistical analyses are presented at genome (Figure 1), chromosome and cluster level (Figure 2). The genome view displays clusters positions and width on different chromosomes; a graphical tree structure enables the user to select a chromosome or a specific cluster of *SF *for further analysis. For each chromosome, a bar plot is given, showing the values (1 - *p*) of windows along sequence-based coordinates (red bars identify significant values). Information about total number of significant clusters of *SF *and of total number of features in significant clusters in the chromosome is also given in a separate panel. For every cluster of *SF*, REEF reports the genomic position (chromosome, start and end bp coordinates) and the number of *SF *and *RF *in significant clusters and in total. The list of features ID/names pertaining to a given cluster can be accessed both as a text file and as a link to the UCSC Genome Browser. In the text file, for each *SF *in the clusters, the corresponding annotation information fields are given, as provided in the input file by the user. These results, which may include gene name, gene symbol, Gene Ontology terms or expression data, can be in turn analyzed by custom-made scripts or by spreadsheets-based methods.

Since REEF exploits UCSC Genome Browser Custom Annotation Tracks facility, clusters and features pertaining to each cluster are visualized as custom tracks, together with standard tracks from UCSC Genome Browser. A "cluster" track shows the position on the chromosome and the width of each given cluster, whereas, a "cluster elements" track shows the position and the width of the different *SF *in the cluster, identified by the name/ID given by the user.

## Results

### Identification of clusters of tissue-specific genes in the human genome

For the sample analysis, we collected a set of 8,959 human genes, mapped on the genomic sequences and associated to good quality expression data in normal tissues. This group of genes was used as a set of *RF*, depicting the gene density along human chromosomes. Among these 8,959 genes, we selected a group of 896 genes expressed prevalently in single tissue, which constituted the set of *SF*. REEF was applied to this dataset, to search for clusters of tissue-specific genes in human chromosomes.

We considered the data of the GNFAtlas2 gene expression survey in 79 human tissues [16]. From the "gnfHumanAtlas2Median table" of the UCSC Genome Browser annotation database hg18 (reporting for each tissue or cell type, median values of expression level measurements in different replicates) we selected expression data pertaining to ten differentiated human tissues under normal conditions (whole brain, lymph node, bone marrow, whole blood, skin, adipocyte, pancreas, skeletal muscle, lung and liver). We used only the subset of expression level measurements belonging to 33,148 Affimetrix_HG_133A probesets, for which quality checked annotation data were available at the GeneAnnot database of Weizmann Institute [17]. GeneAnnot provides a revised and improved annotation of Affymetrix probesets, whose assignment to one or more GeneCards IDs is ranked by sensitivity and specificity scores of probeset/gene matches. We selected only probe-sets reported to be associated to an unique human gene, according to the GeneAnnot database (Release 13). Among them, 16,388, showing either sensitivity and specificity score of 1, were selected. By using GeneALaCart, 15,985 of them were associated to 10,539 EntrezGene IDs. For each of the 3,280 EntrezGene IDs (31%) represented by different probesets, we adopted a jackknifing procedure to integrate expression data in a unique expression vector. For each gene *g*, represented by *N *probesets, we calculated *N *vectors of median values, each for the group of probesets obtained by excluding the *i *probeset (with *i *ranging from 1 to *N*). If Spearman correlation between the calculated vector of median values and of the expression vector of the excluded probeset resulted higher than 0.5, the probeset was discarded. The remained vectors were used to calculate a vector of median values for the gene. In this way we obtained 8,959 unique expression vectors, each for a specific EntrezGene IDs. We then associated a genomic position to these genes, by using the start and end coordinates of the corresponding KnownGene at the UCSC database. For the majority of EntrezGene IDs resulting associated to more than one KnownGene ID, and thus to different genomic positions, the gene width was defined as the maximum genomic region covered by the different KnownGene elements pertaining to the given EntrezGene ID.

These 8,959 human genes were then ranked by their tendency of being regulated in a tissue-dependent manner, such as by their tissue-specificity. For each gene expression vector, we calculated the Shannon Entropy index (H) of tissue-specificity [18] and we selected the 10% of genes apparently most tissue-specific, which constituted the sample of 896 *SF *for subsequent analyses [see additional file 4]. The entire group of 8,959 genes constituted the *RF *sample [see additional files 5 and 6]. By running REEF on these data and selecting window size and shift of 500 and 100 Kb respectively, 44 significant (FDR set to 0.05) clusters of at least 3 tissue-specific genes were identified, accounting for 150 (17%) out of 896 genes included in clusters. Figures 1 and 2 show the graphical display of results produced by REEF.

## Discussion and conclusion

REEF software aims at the identification of genomic regions containing features locally clustered. It can be useful, for example, to find regions enriched in genes with specific expression characteristics, where transcription regulation may operate at chromosomal domain level. As shown in the sample analysis, we identified by REEF clusters of tissue-specific genes in the human genome.

REEF graphical display facilities give several advantages and allow an intuitive and efficient evaluation of analysis results at a glance. Data are presented at different levels: at genome level, all clusters of locally enriched features are shown along the entire genome; at chromosome level, a plot of the quantity (1 - *p-value*) for each window in the selected chromosome is displayed. The chromosome plot outlines regions showing statistically significant local enrichment of the selected features (clusters) and may also reveal regions associated to a statistical score just lower than the threshold, but which may be of special interest since connecting strictly significant clusters in larger regions. Moreover, the integration of REEF with the UCSC Genome Browser Custom Annotation Tracks allows exploration of genomic regions pertaining to selected cluster, giving the possibility to reach all information about the region available at the UCSC Genome Browser database, such as DNA sequence and annotation regarding gene expression data, protein domain and structure information, Gene Ontology, homologous genes and interspecies sequence conservation.

Information on clusters position and content are also given in text format, thus allowing post-processing with custom made scripts or other available software. For instance the user can identify by REEF clusters of a special gene category, defined by analysis of gene expression data, but subsequently analyze obtained lists of clustered genes on the basis of additional different criteria such as Gene Ontology-based function annotation, analyzed to find terms enrichment within clusters.

Due to the very short execution time, subsequent analyses with different settings can be carried out quickly and the genome-wide visualization of results make easy to efficiently compare obtained results.

In order to obtain informative results, search parameters must be chosen appropriately. The "window width" parameter, changing the dimension of the window used to scan the genome by the sliding window approach, should be chosen taking into account the average dimension of considered features, the possible number of features per clusters and the density of features along the considered genomes. We have chosen the 500 Kb width, with about 6 genes per windows expected, since the median value of the span of genes included in our dataset is 26 Kb and the median distance between not overlapping genes is 64 Kb. The "window shift" parameter changes the distance between the starts of adjacent windows in the sliding window algorithm. Both the "window shift" and the "minimum number of features in cluster" parameter influence the FDR calculation, by acting directly on the number of windows on which the statistical test is calculated: a shorter "window shift" makes more stringent the statistical analysis, whereas rising the "minimum number of features in cluster" decreases the stringency.

As said in the Rationale, only scenario-specific programs are available to the researchers for the discovery of local enrichment of features/genes in genomes. The differences in aims and input data of these programs do not allow any direct evaluation of the performances of REEF by comparative algorithms experimentation.

Since REEF can be used on different genomes, to analyze different types of features (genes, DNA motifs, DNA sequences with specific characteristics), it represents an innovative and general tool for detecting the localization of genomic regions of clustered features, thus helping to deepen the knowledge on the architecture and function of genomes. REEF can be useful for studies aiming at evaluating the contribution of chromosomal patterning of co-expressed genes in gene expression regulation, during development and differentiation, with respect to the role of classical gene-specific induction/repression acting on proximal regulatory regions.

## Availability and requirements

Project name: REEF

Project home page: 

Operating system(s): Platform independent

Programming language: Python

Other requirements: python interpreter (version 2.3 or higher), wxPython GUI toolkit

Licence: GNU GPL

Any restrictions to use by non-academics: none

## Authors' contributions

AC, GAD and SB conceived the study and wrote the paper. AC wrote the software, SB carried out the analyses on gene expression data. All authors read and approved the final manuscript.

## Supplementary Material

Additional file 1 REEF multiplatform source code. tarball with REEF source code. For installation instructions see . Click here for file

Additional file 2 The Windows installer. installer for Microsoft Windows, if you choose to install from this package no other software installation is needed. Click here for file

Additional file 3 REEF manual. REEF manual in PDF format. Click here for file

Additional file 4 Selected features. File containing the Selected Features used for the sample analysis (896 tissue-specific genes). For each gene, different columns contain: EntreGeneID, chromosome, bp coordinates of gene start and gene end, gene symbol, gene description, Gene Ontology terms and expression values for the ten considered tissues (whole brain, lymph node, bone marrow, whole blood, skin, adipocyte, pancreas, skeletal muscle, lung and liver). Click here for file

Additional file 5 Reference features. File containing the Reference Features used for the sample analysis (8959 human genes). For each gene, different columns contain: EntreGeneID, chromosome, bp coordinates of gene start and gene end. Click here for file

Additional file 6 Chromosomes length file. The chromosomes length file that must be in the same directory of the reference features file. Click here for file
