# Supplementary material for: REEF: searching REgionally Enriched Features in genomes
Source: BMC Bioinformatics. 2006 Oct 16;7:453. doi: 10.1186/1471-2105-7-453 (PMC1624853; doi:10.1186/1471-2105-7-453)
Supplement: Additional file 3 — REEF manual. REEF manual in PDF format. [file 1471-2105-7-453-S3.pdf]

REEF User Manual

Overview

REEF is aimed at identifying regions of a genome enriched in specific features, as compared with a reference landscape of features density. It takes as input a list of reference features (RF, e.g human genes) mapped on a genome sequence, a list of selected features (SF) among the RF (e.g. human genes specifically expressed in a given tissue) with their genomic positions and the number and the length of the chromosomes in the genome under consideration. It scans the genome using a sliding window approach, and calculates the statistical significance of each windows using the Hypergeometric Distribution and the False Discovery Rate (FDR). Consecutive significant windows form a cluster of regional enriched features. Results can be viewed as plots or dumped to text file for further analysis. The program also allows the user to display the results using the Custom Annotation Tracks facility from the UCSC Genome Browser.

Installation

Binary packages is avaliable for Microsoft Windows, if you choose to install from this package no other software installation is needed. Windows users can download the executable installer, run it and follow the instructions.

The python *source code* is multiplatform, can be run from different operating systems and it requires the following aditional packages to be installed:

- **The python interpreter** (version 2.3 or higher), download it from www.python.org
- **The wxPython gui toolkit**, you can get it form the following web site: www.wxpython.org
- **The SciPy package** tath can be downloaded from http://www.scipy.org/

Once you have installed all the dependencies and downloaded the REEF source code from the download page, extract the directory from the archive an from that directory run the main script called reef.py

Settings

The REEF settings window allows the user to choose the appropriate parameter for the analysis.

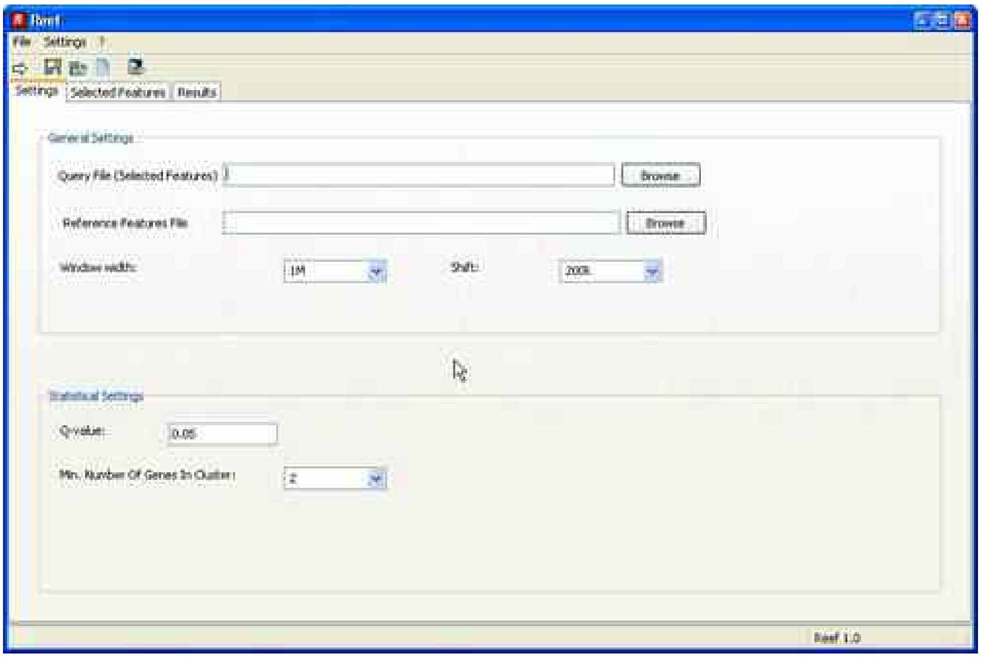

- The *Query File* is the file containing the selected features (e.g. tissue specific genes). It must be a tab separated values text file containing at least four colums: the feature ID and the chromosomal localization in which the feature is found (chromosome, start and end cohordinates, e.g. NM_002291 chr7 107351498 107431040). Optional columns may contain additional description of the feature. A sample query file can be downloaded from REEF web site.
- The *Reference Features File* is the file containing the reference features. It must be a tab separated values file containing four colums: the feature ID and the chromosomal localization in which the feature is found

(chromosome, start and end cohordinates, e.g. NM_002291 chr7 107351498 107431040) *The directory of the reference features file must contain a file with the same name of the reference file but with the .chr extension containing in the first line the organism and in the following lines the name of the chromosomes and their length*; the chomosome length is used for drawing the results and the organism field is used to select the appropriate organism in the UCSC Genome Browser custom annotation view. If the organism is not present in the UCSC Genome Browser database, the visualization of REEF results in the UCSC Genome Browser is not possible. Some allowed organism names are: Human, Chimp, Dog and Cow. See genome.ucsc.edu/cgi-bin/hgGateway to check the avaliable organisms at in the UCSC Genome Browser database. A sample reference file and a sample chromosomes length file for the human genome can be downloaded from REEF web site.

- The *Window width* parameter changes the dimension of the window used to scan the genome by the sliding window approach.
- The *Shift* parameter changes the distance between the starts of adjacent windows in the sliding window algorithm. Obviously the bigger the shift, the less the number of windows considered in the analysis (N). The N parameter influences the FDR calculation: the higher N the most stringent the statistical threshold on the single window.
- The *Q-value threshold* determines the global threshol for significance. Let *S* be the total number of *SF* over the entire genome, *R* the total number of *RF* over the entire genome, and *r* the number of *RF* in a given window (with $R \geq r$ and $S \geq r$). The probability of observing by chance at least *k SF* ($x \geq k$) out of *r RF* in the window is the pointwise significance of the observed numbers of *SF* in the window (p-value, p):

$$p_{x\geq k} = \sum_{x=k}^{r} \frac{\binom{S}{x}\binom{R-S}{r-x}}{\binom{R}{r}}$$

The False Discovery Rate (FDR, Storey and Tibshirani, 2003) is used to circumvent the problem of multiple testing for the genomewide calculation of statistical significance for the observed enrichment in *SF* in a given region. In particular, after sorting windows by *p-values* over the entire genome, *q-values* (FDR) were calculated. *Q* (*q-value*) for each window is defined as *Q=(p*N)/i* , where *p* is the *p-value* of the window, *N* the total number of windows considered and *i* the number of windows with a *p-value* not higher than *p*. Given a global threshold for the genome-wide FDR (e.g. 5%), the number of windows "significantly enriched in *SF*" is determined. The span of the maxi-mum number of adjacent windows showing statistical significance defines the boundaries of one cluster of *SF*.

- The *Minimun Number of Features in Cluster* parameter determines the minimun number of selected features that a window must contain in order to consider the window for further analysis. This parameter influences the FDR calculation: the higher the Minimun Number of Features in Cluster the less stringent the statistical threshold on the single window.

By clicking on the "Start Analysis" button in the toolbar, the program start to run!

Results

The "Selected Features" window contains the list of all the selected features loaded from the input query file.

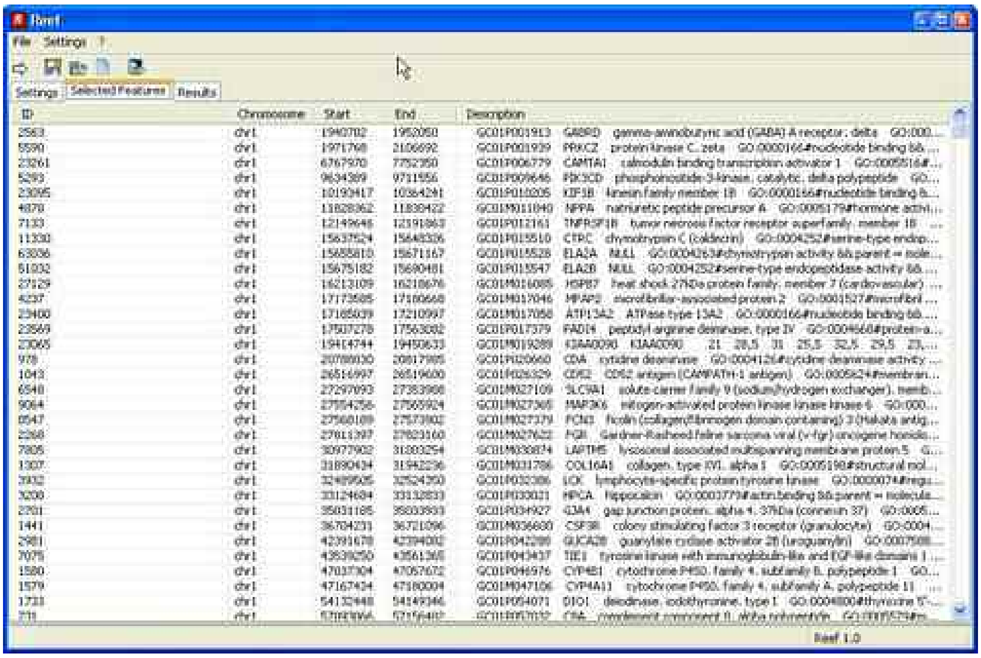

The "Results" window shows the results that are obtained by an analysis. On the left a tree structure allows the user to choose different kind of views. The "genome view" can be accesses by left-clicking on the root of the tree called Chromosomes, it shows all the chromosomes in the genome and the position of clusters of enriched features on the chromosomes. The clusters are represented by red squares; passing the mouse pointer over them the name and the position of the cluster is shown. The "genome statistics" on the bottom rigth shows some statistics about the clusters in the genome:

- *Significant Clusters* shows the total number of significant clusters in the genome
- *Reference Features* shows the total number of reference features in the genome
- *Selected Features* shows the total number of selected features in the genome
- *Reference Features In Clusters* shows the number of reference features contained in clusters

- *Selected Features In Clusters* shows the number of selected features contained in clusters

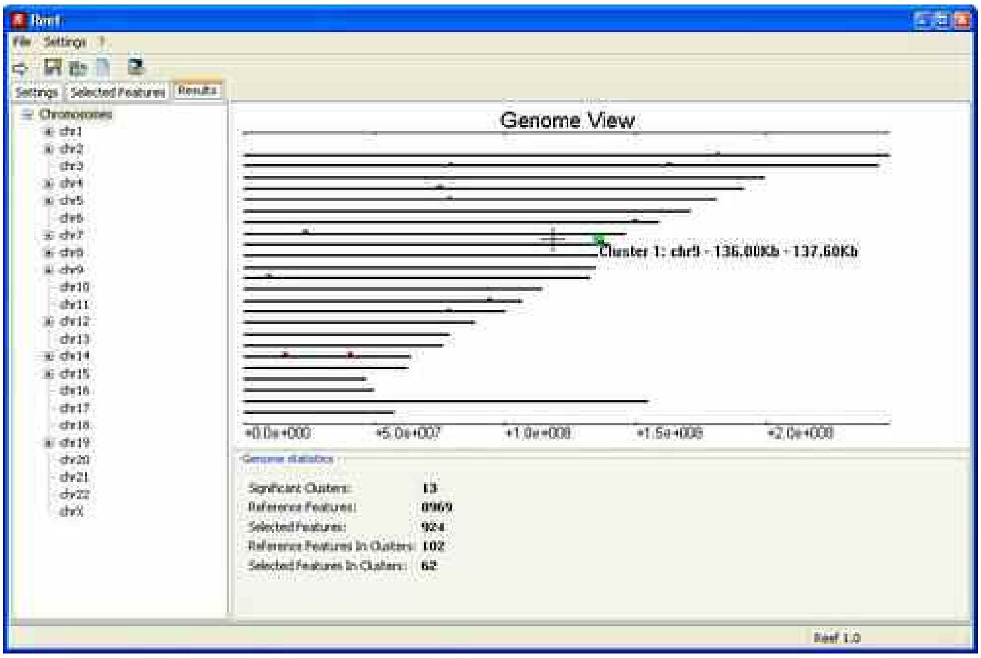

By clicking on a specific chromosome in the tree structure, a bar plot is given, showing the quantity (1 - p) of windows along the sequence-based coordinates of the chromosoem (significant values are represented by red bars). Information about the total number of significant clusters of selected features and of the total number of features in significant clusters in the chromosome are also given in the bottom panel.

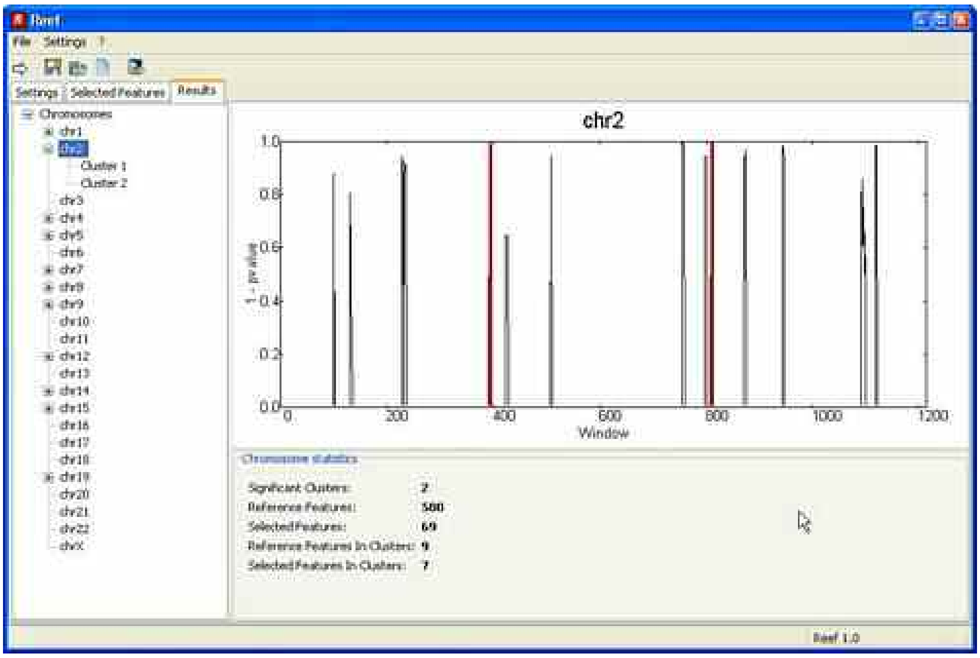

The chromosomes containing significant clusters show + symbol in the tree structure. By clicking on the + symbol the tree structure is expanded in order to show the list of clusters in the chromosome. By clicking on the cluster's name, the plot of the chromosome is shown but only the selected cluster is represented by red bars. The chromosome and the cluster plots are zoomable by left mouse buttom drag; left mouse double click resets the zoom; right mouse click zooms out centered on click location. The botton right subwindow shows the following information about the cluster:

- *Selected Features* shows the number of selected features in the cluster
- *Reference Features* shows the number of reference features in the cluster
- *Worst Window P-Value* shows the p-value of the less significative window in the cluster. Cluster are defined as consecutive significant windows
- *Position* shows the position of the cluster on the chromosome
- *Coverage* shows the extension of the cluster in Megabases
- *UCSC G.B. Custom Tracks Cluster View* is a web link that shows the cluster and it's elements on the UCSC Genome Browser. The features pertaining to the cluster are visualized as custom tracks, together with

standard tracks from UCSC Genome Browser. A "cluster" track shows the chromosome position and the span of each given cluster, whereas a "cluster elements" track shows the position and the span of the different selected features in the cluster, each identified by the name/ID given by the user. In this, way, cluster information can be inspected together with the annotation information available for the considered genome. The user must set the "Cluster" and "Cluster_element" tracks to "full view" on the Genome Browser web page in order to display the custom annotation.

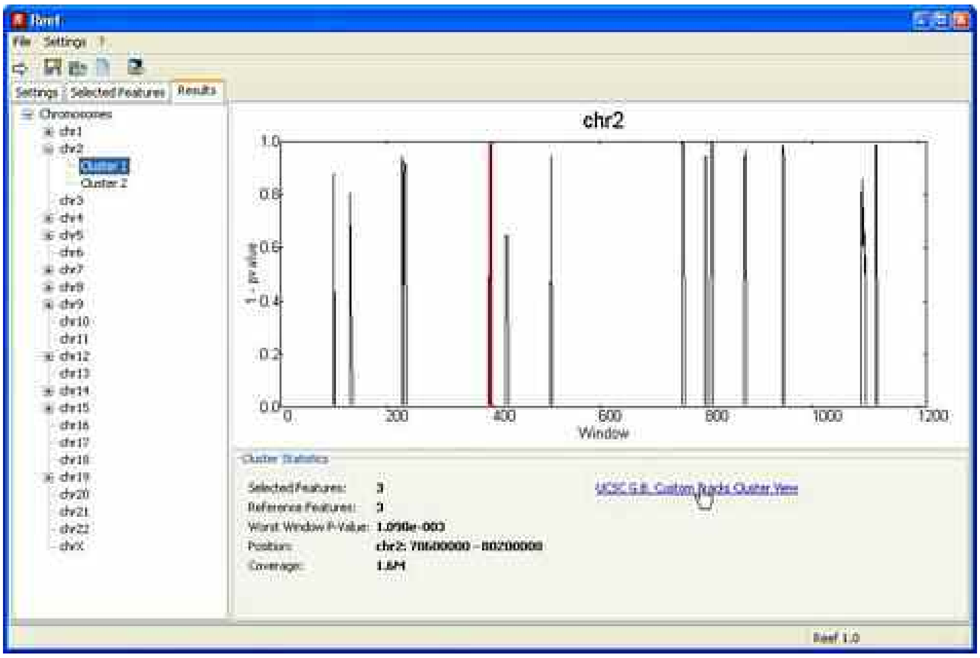

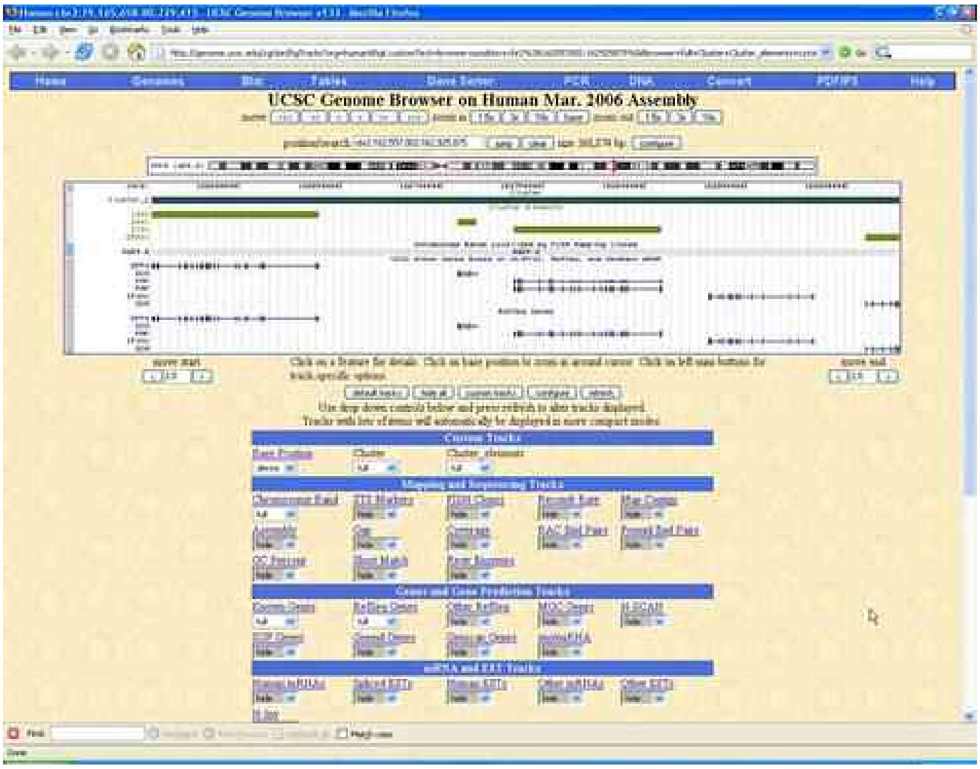

By double clicking on a cluster in the tree structure a new window containing information about the cluster is opened. The window also show the list of selected features Ids pertaining to the cluster and the first part of the feature description provided by the user in the input file.

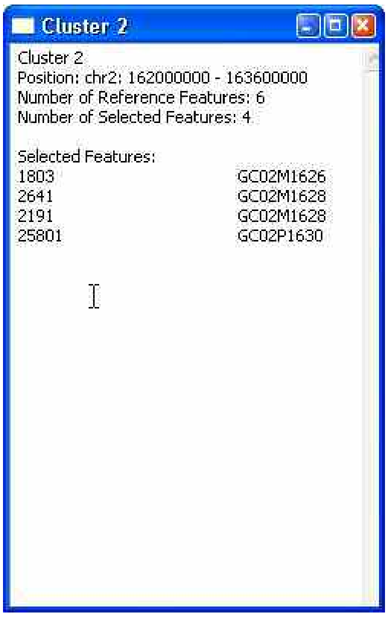

The *"Dump To Text"* button on the toolbar creates a .txt file with information about all clusters. Values are separated by the tab character in order to allow post-processing of the results with custom made scripts or spreadsheets-based programs. The text dump file shows, for every cluster, the list of all the features names/ID, chromosome, start position, end position and annotation information.
